# Cultural competence and associated factors among nurses working in public hospitals of South Wollo zone, Northeast Ethiopia: a multi-center cross-sectional study

**DOI:** 10.1186/s12912-024-02162-x

**Published:** 2024-07-18

**Authors:** Tekola Seid Zeleke, Muluken Amare Wudu, Yemane Eshetu Bekalu, Endalik Birrie Wondifraw, Tarikua Afework Birhanu, Getaw Walle Bazie

**Affiliations:** 1 Jama woreda Health Unit, Jamma, Ethiopia; 2 Department of Pediatrics and Child Health Nursing, College of Medicine and Health Sciences, Wollo University, P. O. Box: 1145, Dessie, 1145 Ethiopia; 3 Department of Public Health, ALKAN Health Sciences and Business College, Dessie, Ethiopia; 4 Department of Epidemiology and Biostatistics, College of Medicine and Health Sciences School of Public Health, Wollo University, Dessie, Ethiopia

**Keywords:** Cultural competence, Nurses, Associated factor, Public hospitals, Northeastern Ethiopia

## Abstract

**Background:**

Enhancing cultural competence stands as a cornerstone in the realm of clinical nursing. Consequently, nurses engaging with culturally diverse communities encounter significant challenges. In Ethiopia, nurses providing care often prioritize physical well-being, the therapeutic journey, and medical interventions, while overlooking the critical cultural dimensions of patient care. Therefore, this study aims to assess the level of cultural competence and its determining factors among nurses employed in public hospitals located in the South Wollo Zone of northeastern Ethiopia.

**Methods:**

A multicenter, institution-based cross-sectional study was conducted, involving 629 nurses employed in public hospitals across northeastern Ethiopia. Participants were selected using a simple random sampling method. Data were gathered using a structured, self-administered English version of the Nurse Cultural Competence Scale Questionnaire (NCCSQ), and subsequently entered into Epi-data 4.6 for analysis. Statistical analysis was performed using SPSS version 26, employing multiple linear regression analysis to identify determining factors.

**Result:**

The participants’ overall mean score for cultural competence was 3.198 [95% CI: 3.161, 3.234]. Specifically, factors such as being a female nurse (β = 0.089, CI: 0.019–0.159), having a diploma level of education (β = -0.084, CI: -0.101 to -0.007), having 11–20 years of work experience (β = 0.412, CI: 0.090–0.815), a 1:15 nurse-to-patient ratio (β = 0.081, CI: 0.010–0.162), experience with caring for culturally and ethnically diverse patients (β = 0.362, CI: 0.248–0.476), comprehensive hospital level (β = 0.699, CI: 0.496–0.903), and attending cultural training (β = 0.002, CI: 0.234–0.931) were predictors of the mean score for cultural competence.

**Conclusion:**

In this study, the level of cultural competence was found to be at a moderate level and need more effort. Factors such as gender, years of work experience, nurse-to-patient ratio, experience in caring for culturally and ethnically diverse patients, hospital level, educational attainment, attendance of cultural training, and the presence of a feedback system for cultural competence were identified as predictors of cultural competence level. Sharing experiences from higher-level hospitals to lower-level ones and strengthening cultural competence training sessions for nurses can significantly enhance cultural competence within clinical settings.

## Background

Cultural competence (CC) is a lifelong process that entails the integration of cultural awareness, cultural knowledge, cultural skills, and cultural sensitivity [1]. Furthermore, it implies that nurses consistently strive to enhance their ability and readiness to work effectively within the cultural context of their clients, whether at the individual, family, or community level [1, 2].

Cultural competence (CC) has garnered significant scholarly interest globally in recent decades, accompanied by heightened commitments to address the needs of culturally diverse clients [1–3]. Consequently, delivering high-quality healthcare as a vital service sector heavily depends on effectively navigating cultural diversity, encompassing differences among patients in gender, race, education, geographical background, language, ethnicity, and other pertinent factors [1–3].

Globally, an estimated twenty-eight million nurses deliver exceptional and profound care to an increasingly diverse population in 2022 [4]. Nonetheless, one of the primary sources of health inequalities worldwide stems from cultural diversity, leading to poorer patient outcomes and diminished satisfaction with care [4, 5].

Client empowerment, perceived respect from clients, increased compliance with treatment regimens, and improved outcomes are among the benefits of culturally competent care [6]. However, a systematic scoping review conducted in Canada, New Zealand, Australia, and the United States revealed that 31% of participants experienced insufficient culturally competent nursing care [7]. Furthermore, studies conducted in the USA, Canada, various countries in Europe, Austria, India, and China have indicated a moderate level of cultural competence (CC) in nursing care, suggesting a need for further improvement [6, 8–12]. Similarly, studies conducted in Ethiopia have shown that the level of CC among nurses ranges from low to moderate [13–16].

Sociodemographic factors, organizational factors (such as lack of organizational support, participation in in-service training, hours of continuing education related to cultural nursing care, nurse-to-patient ratio, frequency of caring for clients from culturally and ethnically diverse backgrounds, and the presence of a feedback system in health facilities), language barriers, cultural differences, and limitations related to healthcare providers (including trained interpreter and translator availability, lack of knowledge of the patient’s culture, insufficient multicultural nursing workforce, and shortages of nursing staff) have all been associated with the level of cultural competence [8–16].

Cultural competency was formally recognized and integrated into the scope and standards of nursing practice by the American Nursing Association in 2016 [17]. Furthermore, the American Academy of Nursing (AAN) has developed universally applicable guidelines for delivering culturally competent care, serving as a valuable resource for nurses across various specialties. However, despite these efforts, the implementation of cultural competence in nursing practice remains limited and calls for further action [18]. Over the last five years, the Ethiopian Ministry of Health has implemented the Compassionate, Respectful, and Caring initiative to promote patient-focused care, with cultural-based care included as a subcomponent of the protocol [19, 20].

Despite the imperative for healthcare providers to be culturally competent in a multicultural society, there remains a scarcity of studies on the cultural competence of nurses in Ethiopia [13–16], particularly with no documented research in the study area. Moreover, in contrast to previous studies, the current study spans across multiple hospitals and encompasses a wide geographical area, facilitating a more comprehensive inference. Therefore, this study addresses the knowledge gap regarding the status of CC and its associated factors among nurses in Northeastern Ethiopia. Consequently, healthcare managers and nurses can identify deficiencies in cultural competence nursing care, prompting regional and national health bureaus to implement measures aimed at enhancing cultural competence among care providers and ultimately ensuring the delivery of quality care.

## Methods and materials

### Study area, period, and design

A multi-centered hospital-based cross-sectional study design was implemented in the South Wollo Zone, situated in northeastern Ethiopia, spanning from November 2023 to December 2023. This zone is an administrative district within the eastern Amhara region of northeastern Ethiopia, with Dessie serving as its capital city. Within this zone, there are 14 government hospitals employing 1150 nurses, along with an additional 5 private general hospitals. However, despite the presence of these healthcare facilities, the public hospitals in the South Wollo Zone are tasked with serving a population exceeding 10 million people. As a result, the public hospitals in the South Wollo Zone cater to a diverse range of individuals, including lowlanders, highlanders, rural dwellers, urban residents, farmers, and intellectuals. Furthermore, the patient population comprises followers of various religious beliefs, including Muslims, Orthodox Christians, Protestants, and practitioners of local religions.

### Study populations

All nurses employed at the public hospitals in the South Wollo Zone, northeastern Ethiopia, were considered as the source of population. However, only a randomly selected nurses working at selected public hospitals in the South Wollo Zone, northeastern Ethiopia, were included in the actual data and considered as study population.

### Eligibility criteria

Nurses working at selected public hospitals in the South Wollo Zone, available during the data collection period, and possessing a minimum of six months of work experience were included in the study. However, nurses who fell ill during the data collection period were excluded from the study.

### Sample size determinations

The sample size was determined using a formula for a single population mean,

$$\text{n}=\frac{(\text{Z}\alpha/2)^{2}\;\text{x}(\sigma)^{2}}{(\text{d})^{2}}$$

considering the following assumptions: a confidence level of 95% and a margin of error(d) was nearly 0.05 based on the calculation of the previous study [14], the standard deviation ((σ = 0.61), *n* = 543, SE = σ/√n, 0.61/ √543 = 0.026177606 and σ^2^ = variance of the mean). Therefore, calculated margin of error (d) → zα/2*SE = which is 0.026177606 *1.96 = 0.0513 ≈ 0.05. Therefore, the final sample size was calculated as follows:

$$\text{n}=\frac{(1.96)^{2}\ast (0.61)^{2}}{(0.05)^{2}}$$

was 572. so, the final sample size by adding 10% (57) of the non-response rate was 629.

### Sampling technique and procedures

Five out of the fourteen government hospitals in the South Wollo Zone were selected using a simple random method, employing a lottery technique. These hospitals, namely Dessie Comprehensive Specialized Hospital (525 nurses), Kombolicha General Hospital (65 nurses), Akesta Referral Hospital (70 nurses), Empress Zewuditu General Hospital (65 nurses), and Mekaneselam General Hospital (52 nurses), collectively house a total of 777 nurses. Subsequently, lists of potential study subjects were distributed among these randomly selected hospitals using proportional allocation. Consequently, the number of nurses selected from each hospital was as follows: Dessie Comprehensive Specialized Hospital (425 nurses), Kombolicha General Hospital (53 nurses), Empress Zewuditu General Hospital (53 nurses), Akesta Referral Hospital (56 nurses), and Mekaneselam General Hospital (42 nurses). Moreover, gender and ward (working unit) representation were considered in addition to the client load in each hospital. After determining the sample size, a simple random sampling technique was utilized to choose the final study participants from the prepared sampling frame, which comprised the list of nurses employed at public hospitals in the South Wollo Zone. Subsequently, the initial study subject was selected via lottery methods, followed by selecting every 2 k^th^ intervals until the required sample size was achieved in each hospital. The sampling frame for the study participants consisted of the human resource department list, which includes nurses and their respective departments. Instead of nurses who fell ill during data collection, the next available participant was selected to replace them. Since we had coded all participants, the selection was made in the same setting (hospital).

### Operational definitions

The level of overall cultural competence was determined by categorizing it based on the mean score of the Cultural Competence Questionnaire for Nurses (CCQN) scale score across four domains and the Cultural Competence Assessment Scale. This scale measures the score item by item, with the rank indicating the highest level achieved: a score of 1 denotes no activity on that criterion, while a score of 5 indicates meeting the benchmark standard. The final score is obtained by summing up the items [14, 21, 22]. Furthermore, each subscale of cultural competence—cultural awareness, cultural knowledge, cultural sensitivity, and cultural skill—was categorized based on the mean score of each subscale into four categories: low-level cultural competence (mean score = 1–1.80), low to moderate level cultural competence (mean score = 1.81–2.60), moderate level cultural competence (mean score = 2.61–3.40), and high-level cultural competence (mean score > 3.41) [13, 15, 21, 22].

### Dependent variables

Level of Cultural competence.

### Independent variables

Socio-demographic factors include multicultural residence, multilingualism, age, marital status, gender, and educational attainment of the nurses. Nurse-related factors encompass self-perception regarding cultural competence, participation in cultural diversity training, years of work experience, experience in caring for clients from culturally and ethnically diverse backgrounds, and employment in various levels of healthcare settings. Furthermore, organizational-related factors involve the use of healthcare interpreters, nurse-to-patient ratio, availability of a feedback system, and organizational support.

### Data collection and procedures

The data collection tool used was the ‘Nurse Cultural Competence Scale Questionnaire (NCCSQ),’ adapted from previous studies [14, 15], comprising four sections. The first three sections encompass socio-demographic variables, nurse-related factors, and organizational-related variables, respectively, followed by an assessment of cultural competence levels. Data collection was conducted using a structured and self-administered English version questionnaire.

The NCCSQ comprises a total of 41 items, distributed across cultural awareness (11 items), cultural knowledge (8 items), cultural sensitivity (8 items), and cultural skills (14 items). Each item employs a five-point Likert scale to gauge the participant’s response, ranging from ‘1 = totally disagree’ to ‘5 = 100% agree.’ For data collection and supervision, five data collectors holding a BSc degree in nursing, along with two supervisors holding an MSc in nursing, were recruited. Additionally, internal content validity was ensured by involving five nurse experts from various universities across the country. The content validity ratio (CVR) and content validity index (CVI) were calculated, yielding values of 0.81 and 0.84, respectively, affirming the instrument’s validity, as reported in previous studies [14, 15].

### Data quality control

Two days of training were provided for both data collectors and supervisors, covering topics such as confidentiality, the objectives of the study, and techniques for administering the questionnaire. Supervisors ensured the completeness of the data during the data collection process. A pretest was conducted at Haik Primary Hospital, involving 5% (31) of the sample size. In this study, Cronbach’s alpha coefficients for cultural awareness, cultural knowledge, cultural sensitivity, and cultural skill were found to be 0.79, 0.91, 0.82, and 0.84, respectively. The reliability of the cultural competence tool was determined to be 0.83, indicating sufficient reliability.

### Data analysis

The data were coded, entered, and cleaned using Epi-data version 4.2 and subsequently exported to SPSS Version 26 for analysis. Descriptive statistics, including mean, standard deviations, and frequencies, were employed to characterize the study population, presented through both textual descriptions and tables.

Prior to conducting univariate linear regression analysis, several assumptions were verified. Linearity was confirmed through scatter plots, demonstrating a linear relationship between the independent and dependent variables. Normality of residuals was assessed visually using p-p plots, indicating a normal distribution with residuals aligning closely to the diagonal line. Additionally, Shapiro-Wilk test yielded a p-value of 0.123, further supporting normality. Multicollinearity was addressed by examining variance inflation factors (VIF), ranging from 1 to 3.9, indicating no significant multicollinearity. Autocorrelation was assessed using the Durbin-Watson test, yielding a value of 1.867, suggesting no autocorrelation. Homoscedasticity was evaluated through scatter plots, demonstrating a consistent spread of residuals. Finally, influential outliers were examined using Cook’s distance, with no values exceeding one. Following these checks, simple linear regression was conducted to identify potential factors for inclusion in multiple linear regression, with a significance level set at *p* < 0.25. Factors meeting this criterion were then included in the multiple linear regression analysis, with statistical significance determined at a p-value < 0.05.

## Result

### Socio-demographic characteristics of the participants

A total of 600 participants were enrolled in the study, yielding a response rate of 95.4%. Among the participants, 339 (56.5%) were female, and 217 (36.2%) were aged between 36 and 40 years. The mean age of the participants was 33.09 years, with a standard deviation of ± 5.881. Furthermore, the majority of participants, 575 (95.6%), identified as Amhara ethnicity, followed by 318 (53%) identifying as Orthodox in their religion. Additionally, 389 participants (77.3%) held bachelor’s degrees **(see** Table 1**)**.

**Table 1:** Socio-demographic and socio-economic characteristics of nurses working in public hospitals of northeastern Ethiopia,2024

Variables	Frequency	Percent	Mean and standard deviation
**Age of respondents**			33.09 ± 5.881
21–25 years	72	12	
26–30 years	160	26.7	
31–35 years	129	21.5	
36–40 years	217	36.2	
41–60 years	22	3.7	
**Sex**			
Male	261	43.5	
Female	339	56.5	
**Marital status**			
Married	347	57.8	
Single	350	41.7	
Divorced	3	0.5	
**Ethnicity**			
Amhara	575	95.8	
Others (Oromo, Tigray, and Wolaita)	25	4.2	
**Religious status**			
Orthodox	318	53	
Muslim	248	41.3	
Protestant	34	5.7	
**Educational status**			
Diploma	21	3.5	
Bachler degree	389	64.8	
Master’s degree	190	31.7	

### Work and organizational related characteristics

A substantial portion of participants, totaling 304 (50.7%), possessed work experience ranging from 11 to 20 years. Furthermore, the majority, comprising 529 (88.2%), were staff nurses. A total of 317 (52.8%) were multilingual, and 320 (53.3%) had previously worked at the comprehensive hospital level. Additionally, a significant proportion, accounting for 537 (89.5%), had experience in caring for multicultural patients. Out of 600 participants, 261 reported their hospital’s nurse-to-patient ratio. However, the rest did not know their hospital’s nurse-to-patient ratio. Moreover, a mere 6 (1%) had received cultural training. Moreover, the vast majority, totaling 555 (92.5%) and 562 (93.7%) participants respectively, reported the absence of a feedback system or organizational support for cultural competence (see Table 2**)**.

**Table 2:** Work and organizational related characteristics of nurses working in public hospitals of northeastern Ethiopia, 2024

Variables	Frequency	Percent	Mean and standard deviation
**Work experience**			10.46 ± 6 yrs
1–5 years	187	31.2	
6–10 years	95	15.8	
11–20 years	304	50.7	
> 20 years	14	2.3	
**Being multilingual**			
Yes	317	52.8	
No	283	47.2	
**Current role**			
Staff nurse	529	88.2	
Head nurse	60	10.8	
Matron nurse	8	1	
**Facility level**			
Comprehensive specialized Hospital	320	53.3	
General Hospital	280	46.7	
**Previous working facility level**			
Comprehensive specialized Hospital	90	15	
General Hospital	223	37.2	
Primary Hospital	71	11.8	
Health center	216	36	
**Experience of caring multicultural patient**			
Yes	537	89.5	
No	63	10.5	
**Have received cultural training**			
Yes	6	1	
No	594	99	
**Presence of feedback system for cultural competence**			
Yes	45	2.5	
No	555	92.5	
**Presence of organizational support for cultural competence**			
Yes	38	6.3	
No	562	93.7	
**Nurse-to-patient ratio**			
1:7	261	43.5	
1:10	199	33.2	
1:15	140	23.3	

### Level of cultural competence

The overall mean score of cultural competence among participants was 3.198 ± 0.456 SD [95% CI: 3.161, 3.234], indicating a moderate level of cultural competence. Among the subdomains, cultural awareness achieved the highest mean score of 3.61 ± 0.52 SD. Conversely, cultural knowledge obtained the lowest mean score, standing at 2.83 ± 0.64 SD. The mean score for cultural sensitivity was 3.38 ± 0.65 SD, also reflecting a moderate level of cultural competence. Lastly, cultural skill garnered a mean score of 2.97 ± 0.60 SD, similarly indicating a moderate level of cultural competence **(**Table 3**)**.

**Table 3:** The level of cultural competence among nurses working in public hospitals of northeastern Ethiopia,2024 (*n* = 600)

Variables	Mean item score of the domain		Level of cultural competence
	M ± SD	Range	
Cultural awareness	3.61 ± 0.52	1.82–4.73	High level
Cultural knowledge	2.83 ± 0.64	1.50–4.63	Moderate level
Cultural sensitivity	3.38 ± 0.65	1.88–4.88	Moderate level
Cultural skill	2.97 ± 0.60	1.93–4.57	Moderate level
Cultural competence	3.198 ± 0.456	2.16–4.42	Moderate level

### Factors associated with the level of cultural competence

In the simple linear regression model, variables including gender, age, years of work experience, nurse-to-patient ratio, previous experience working in health institutions at different levels, educational level, experience in caring for culturally and ethnically diverse patients, and the existence of a feedback system within health institutions were found to be associated with cultural competence at a p-value < 0.25. Consequently, these variables were selected for inclusion in the multiple linear regression analysis.

In multiple linear regression analysis, gender, years of work experience, nurse-to-patient ratio, experience in caring for culturally and ethnically diverse patients, level of health facility, educational status, and participation in cultural training were identified as influential factors affecting the level of cultural competence **(**Table 4**)**.

**Table 4:** Factors associated with the level of cultural competence among nurses working in public hospitals of northeastern Ethiopia,2024 (*n* = 600)

Variables	Unstandardized β	Standardized β	t	*p*-value	95% CI		VIF
					Lower bound	Upper bound	
Age	− 0.005	− 0.069	− 0.049	0.683	− 0.031	0.020	2.148
Work experience	**0.412**	0.205	2.008	**0.045**	**0.09**	**0.815**	1.546
Sex(females)	**0.089**	0.097	2.487	**0.013**	**0.019**	**0.159**	1.158
Educational status	**− 0.084**	− 0.109	-2.237	**0.026**	**− 0.101**	**− 0.007**	1.844
Being multilingual	− 0.009	− 0.010	− 0.236	0.814	− 0.085	0.066	1.363
Current role	0.027	0.023	0.609	0.543	− 0.080	0.115	1.136
Current facility level (comprehensive hospital)	**0.699**	0.765	6.750	**0.000**	**0.496**	**0.903**	1.985
Experiencing of caring multicultural patient	**0.362**	0.253	6.253	**0.000**	**0.248**	**0.476**	1.276
Have taken cultural training	**0.002**	0.723	5.268	**0.000**	**0.234**	**0.931**	1.559
Presence of feedback system for cultural competence	− 0.082	− 0.047	-1.193	0.234	0.053	0.822	1.217
Presence of \organization support for cultural competence	0.126	0.067	1.614	0.107	− 0.027	0.279	1.339
Nurse-to-patient ratio	**0.081**	0.141	1.974	**0.049**	**0.010**	**0.162**	3.920

Bold text in the unstandardized B and 95% CI represent the associated facors and in *p*-value indicates stastistically significant factors

As work experience progressed by a year, there was a corresponding increase of 0.412 units in the mean score of cultural competence (β = 0.412, CI = 0.09–0.815). Likewise, with each advancement in education level among nurses, there was a decrease in the mean score of cultural competence by -0.084 (β = -0.084, CI = -0.101, -0.007). Additionally, female nurses demonstrated an increase in the mean score of cultural competence by 0.089 units (β = 0.089, CI = 0.019–0.159) compared to their male counterparts. Furthermore, for each incremental increase in the nurse-to-patient ratio, the cultural competence mean score rose by 0.081 units (β = 0.081, CI = 0.010–0.162). With each increment in the level of hospital, there was an increment of 0.699 units in the mean score of cultural competence (β = 0.699, CI = 0.496–0.903). Furthermore, for every advancement in experience in caring for multicultural patients by one step, there was an increase of 0.362 units in the mean score of cultural competencies (β = 0.362, CI = 0.248–0.476). Lastly, for each additional session of cultural competence training received, there was a marginal increase of 0.002 units in the mean score of cultural competencies (β = 0.002, CI = 0.234–0.931).

### Model fitness

The overall goodness of fit of the model was ascertained by ANOVA with a significant F –test equation was found (F) = 13.308, p- value = < 0.0010. Moreover, the model summary was ascertained with R square = 0.642, resulting 64.2% of the variation in cultural competence of nurses was explained by independent variable in the model.

## Discussion

Ethiopia, with its rich tapestry of ethnic and cultural diversity, presents a frequent encounter for nurses in public hospitals. This study provides invaluable insights into the cultural competence levels and associated factors among Ethiopian nurses in these settings. Overall, the participants exhibited moderate level of cultural competence. Furthermore, gender, years of work experience, nurse-to-patient ratio, experience in caring for culturally and ethnically diverse patients, level of healthcare facility, educational attainment, participation in cultural training, and the existence of a feedback system for cultural competence emerged as predictors of the cultural competence level.

In the current study, the mean score of cultural competence was moderate level. This is comparable with findings from Italia [23], Austria [10], India [11], China [12], Taiwan [24], and Korea [25]. Furthermore, it is in line with findings in Assossa western Ethiopia [15], and Amhara region northwestern Ethiopia [14]. However, the current study finding is higher than studies conducted in Indonesia [26], South Africa [27], and southwest Ethiopia [13]. The differences in findings could potentially be attributed to variations in hospital levels, sample sizes, nurse and patient awareness of cultural diversity, and trends in patient-centered care among the studies. Additionally, experience with national policies, programs, and proclamations concerning patient rights and medicolegal issues, as well as measures taken, may also have contributed to these differences. Similarly, the findings of this study are lower than those reported in studies conducted in Canada [6] and Taiwan [28]. This variance could potentially be attributed to differences in sample size, nurse and patient awareness of cultural diversity, clinical settings, trends in patient-centered care, and experience with national policies, programs, and proclamations concerning patient rights and medicolegal issues.

In this study, the cultural competence subscale with the highest score was cultural awareness, a trend consistent with findings from the USA [8], Austria [10], Taiwan [24, 28], Indonesia [26], and Assossa in western Ethiopia [15]. However, cultural knowledge emerged as the subscale with the lowest score, mirroring a study conducted in Taiwan [24]. One possible explanation could be that participants lacked sufficient training in health-related cultural knowledge and cultural care nursing practices.

The current study unveiled that with each additional female nurse, there was an increment of 0.097 units in the mean score of cultural competence. This finding aligns with results reported in Europe [9]. One plausible explanation could be attributed to the experience female nurses gain in caring for various age groups within their families, ranging from infants to elderly individuals. This diverse caregiving background may contribute to their development as culturally competent care providers within healthcare facilities [6, 8, 9]. Moreover, females tend to be relatively more patient by nature, which may enable them to be more aware of and apply cultural competence skills more effectively than males. This suggests that appointing female nurses as mentors for cultural competence in nursing care settings enhances overall cultural competence. Consequently, this improvement can positively impact patient satisfaction and the quality of care delivered.

Furthermore, with each incremental increase in the nurse-to-patient ratio, there was a corresponding rise of 0.141 units in the cultural competence mean score. This trend is consistent with findings observed in Assossa, western Ethiopia [15]. One potential explanation for this association is that as the nurse-patient ratio increases, nurses are exposed more frequently to providing care for culturally diverse patients. Consequently, over time, they may integrate cultural knowledge and skills with heightened awareness, curiosity, and sensitivity towards their patients’ cultural beliefs.

Similarly, as the nurses’ level of education decreased by one level, the mean score of cultural competence decreased by 0.109 units. This finding is consistent with studies conducted in China [12], Italy [23], Austria [10], Saudi Arabia [29], and Ethiopia [15]. One possible explanation for this trend could be that nurses with a master’s level of education are more likely to have received training in transcultural nursing care as part of the foundational curriculum in their post-graduate programs. Additionally, they may be more inclined towards pursuing advanced training and engaging in self-directed learning compared to nurses with diplomas or undergraduate degrees [15]. This suggests that cultural competence training should be integrated into diploma and undergraduate level nursing curricula, and also provided as part of in-service training before nurses enter the workforce.

As work experience increases by a year, the mean score of cultural competence also increases by 0.205 units. This finding aligns with results from a study conducted in China [12]. One possible explanation for this trend could be attributed to the accumulation of knowledge about different cultures through exposure to various media sources and frequent residence in or visits to culturally diverse locations. Moreover, with more years of work experience, nurses are increasingly exposed to culturally diverse patients, their families, and fellow staff members. This exposure enhances their ability to provide culturally competent care. This suggests that pairing more experienced nurses with junior colleagues in clinical settings can facilitate the sharing of knowledge and experience. Through mentorship and coaching, experienced nurses can help junior staff members develop their skills and competencies. Ultimately, this collaborative approach can contribute to improvements in patient satisfaction and the overall quality of care delivered.

Those who worked at the comprehensive specialized hospital level exhibited a 0.765-unit increase in the mean score of cultural competencies compared to those working at the general hospital level. This finding aligns with a study conducted in India [11]. This could be attributed to several factors unique to higher-level hospitals, such as comprehensive specialized hospitals. These institutions typically offer a wider range of specialty services, attract culturally diverse and multilingual patients referred from lower-level hospitals, employ staff members from diverse cultural backgrounds, and are often located in urban areas with diverse populations. Consequently, nurses working in such settings are likely to experience greater cultural exposure, leading to increased cultural awareness, knowledge, sensitivity, and skills.

As the experience of caring for multicultural patients increases, the mean score of cultural competencies also increases by 0.141 unit compared to their counterparts. This trend is consistent with studies conducted in Austria [10], India [11], China [12], Taiwan [24], Saudi Arabia [29], and Ethiopia [15]. This could be attributed to the fact that the more frequently nurses provide care to patients from diverse cultural backgrounds, the more knowledgeable and sensitive they become to culturally competent care practices.

Those who had received cultural competence training showed a significant increase of 0.723 units in their mean score of cultural competencies compared to their counterparts. This finding is consistent with studies conducted in Austria [10], India [11], China [12], Taiwan [24], Saudi Arabia [29], and Ethiopia [16]. One possible explanation for this association is that the more training nurses receive in cultural competence, the more frequently they practice culturally competent behaviors. This highlights the importance of providing cultural competence training not only as part of on-the-job training but also prior to joining the healthcare industry (through in-service training) and incorporating it into nursing education curricula.

### Strengths and limitations of the study

This study utilized a standardized tool and included the maximum possible sample size to draw meaningful inferences. However, it’s important to note that the data were collected through self-administered questionnaire, which may introduce respondent bias. Additionally, because of the cross-sectional study design, we cannot establish a cause-and-effect relationship. Additionally, this study solely reported quantitative results, while exploring the facilitators and barriers of cultural competence would benefit from qualitative inquiry.

## Conclusions and recommendations

In this study, the level of cultural competence among nurses in the study area was found to be at a moderate level. Furthermore, factors such as gender, years of work experience, nurse-to-patient ratio, experience in caring for culturally and ethnically diverse patients, level of hospital, educational attainment, and participation in cultural training were identified as influencing the level of cultural competence.

For the zonal health unit, it is imperative to prioritize and enhance the cultural competence levels among lower-level hospitals (general and primary hospitals). This can be achieved by implementing targeted cultural training programs and fostering organizational support to ensure effective integration of cultural competence practices into patient care. Similarly, for local hospitals and nurse managers, it is essential to prioritize cultural training for nurses. Additionally, they should encourage the assignment of nurses based on their work experience, gender, and nurse-patient ratio to optimize cultural competence in patient care. Nurses, particularly female and more experienced ones, should take on mentorship roles to coach junior and male nurses on cultural competence skills and awareness through peer education initiatives. Moreover, higher education institutions offering undergraduate and postgraduate nursing programs need to allocate adequate time and resources to incorporate cultural theories and provide pre-service cultural competence training for aspiring nurses. Therefore, future researchers should consider employing mixed-methods studies to gain a more comprehensive understanding of cultural competence in nursing.

### Implication for nursing practice

The regional health bureau and comprehensive hospitals need to include cultural competence care training sessions in their continuous professional development (CPD) programs. Additionally, higher education institutions should provide intensive in-service training and incorporate cultural competence into the nursing curriculum. The Ministry of Health should prepare guidelines on cultural competence for care providers, including nurses, and consider its implementation as an indicator of quality of care.

## Data Availability

All the data used for analysis are available from the corresponding author on reasonable request.

## Abbreviations

CC: Cultural competence
NCCSQ: Nurse Cultural Competence Scale Questionnaire
SD: Standard Deviation
